# Stalk architecture, cell wall composition, and QTL underlying high stalk flexibility for improved lodging resistance in maize

**DOI:** 10.1186/s12870-020-02728-2

**Published:** 2020-11-11

**Authors:** Xiaqing Wang, Zi Shi, Ruyang Zhang, Xuan Sun, Jidong Wang, Shuai Wang, Ying Zhang, Yanxin Zhao, Aiguo Su, Chunhui Li, Ronghuan Wang, Yunxia Zhang, Shuaishuai Wang, Yuandong Wang, Wei Song, Jiuran Zhao

**Affiliations:** 1 Beijing Key Laboratory of Maize DNA Fingerprinting and Molecular Breeding, Maize Research Center, Beijing Academy of Agriculture & Forestry Sciences (BAAFS), Shuguang Garden Middle Road No. 9, Haidian District, Beijing, 100097 China; 2 Beijing Key Lab of Digital Plant, Beijing Research Center for Information Technology in Agriculture, Beijing Academy of Agriculture and Forestry Sciences (BAAFS), Shuguang Garden Middle Road No. 11, Haidian District, Beijing, 100097 China

**Keywords:** Maize, Lodging, Stalk flexibility, BSA, KASP

## Abstract

**Background:**

Stalk fracture caused by strong wind can severely reduce yields in maize. Stalks with higher stiffness and flexibility will exhibit stronger lodging resistance. However, stalk flexibility is rarely studied in maize. Stalk fracture of the internode above the ear before tasseling will result in the lack of tassel and pollen, which is devastating for pollination in seed production. In this study, we focused on stalk lodging before tasseling in two maize inbred lines, JING724 and its improved line JING724A1 and their F_2:3_ population.

**Results:**

JING724A1 showed a larger stalk fracture angle than JING724, indicating higher flexibility. In addition, compared to JING724, JING724A1 also had longer and thicker stalks, with a conical, frustum-shaped internode above the ear. Microscopy and X-ray microcomputed tomography of the internal stalk architecture revealed that JING724A1 had more vascular bundles and thicker sclerenchyma tissue. Furthermore, total soluble sugar content of JING724A1, especially the glucose component, was substantially higher than in JING724. Using an F_2:3_ population derived from a JING724 and JING724A1 cross, we performed bulk segregant analysis for stalk fracture angle and detected one QTL located on Chr3: 14.00–19.28 Mb. Through transcriptome data analysis and ∆ (SNP-index), we identified two candidate genes significantly associated with high stalk fracture angle, which encode a RING/U-box superfamily protein (Zm00001d039769) and a MADS-box transcription factor 54 (Zm00001d039913), respectively. Two KASP markers designed from these two candidate genes also showed significant correlations with stalk fracture angle.

**Conclusions:**

The internode shape and glucose content are possibly correlated with stalk flexibility in maize. Two genes in the detected QTL are potentially associated with stalk fracture angle. These novel phenotypes and associated loci will provide a theoretical foundation for understanding the genetic mechanisms of lodging, and facilitate the selection of maize varieties with improved flexibility and robust lodging resistance.

**Supplementary Information:**

The online version contains supplementary material available at 10.1186/s12870-020-02728-2.

## Background

In grain crops such as maize, lodging disrupts the optimal spatial distribution of the plants, resulting in interference with water and nutrient transport, and ultimately leading to less efficient mechanical harvesting and reduced yields [1]. Root lodging and stalk fracture are two major types of lodging [2], with stalk fracture in the early stage of tasseling causing devastating effects to the tassel and/or the ear, and resulting in significant yield loss.

Stalk fracture happens when the pressure from wind forces the stalk past its maximum bearable bending angle, or stalk fracture angle. Thus, the stalk fracture angle can represent the wind resistance of the stalk. Under the same environmental conditions, the greater the bending angle for a plant, the greater the flexibility, and thus the better wind resistance. In contrast, stalks with extremely poor flexibility typically exhibit brittleness. To date, a number of fragile stalk mutants have been reported [3–8], and they share similar phenotypes in the stalk architecture, vascular bundles, and cell wall composition.

Stalk thickness can significantly enhance resistance to fracture [8–10]. For example, rice with increased basal culm diameter and stiffness showed improved resistance to lodging [11]. However, the small stalk diameter with low stiffness in the maize brittle mutant line *bk4* made it susceptible to strong wind and mechanical damage [8]. Furthermore, the shape and number of vascular bundles also affects stalk stiffness and lodging resistance. For example, several vascular bundle phenotypes have been shown to contribute to stalk brittleness, such as malformed vascular bundles in the maize *bk4* mutant, the reduced number of vascular bundles in the *bk2* maize brittle stalk mutant [4, 8], and the insufficient thickness of sclerenchyma in *bc1*, *bc5,* and *bk2* mutants [3–5].

In addition, cell wall components have also been associated with stalk fragility in rice, maize, and barley [3–8, 12], and monosaccharide profiles are reported to vary between mutants and wild-type lines. In the rice *cwa1* and barley *fs2* mutants, only cellulose was reduced [6, 12], whereas cellulose, hemicellulose, and lignin were all reduced in the rice *bc5* mutant compared to wild type [5]. In maize *bk4* mutants, the contents of lignin and cellulose, as well as glucose and mannose, were all reduced [8]. In rice *bc1*, sorghum *sbbc1,* and maize *bk2* mutants, elevated levels of lignin and decreased cellulose and hemicellulose were observed [3, 4, 13]. Moreover, the amount of cellulose and hemicellulose were inversely related in rice *bc6* mutants [7].

In addition to brittleness, extreme stalk flexibility can also result in stalk lodging because the stalk is not rigid enough to provide sufficient support. Previous studies have shown that the *fc*1 mutant in rice and *irx4* mutant in *Arabidopsis* caused lodging [14, 15]. Rice *FC1*, encoding cinnamyl alcohol-dehydrogenase, was found to be primarily expressed in the secondary cell walls of sclerenchyma cells and vascular bundle regions. In *FC1* loss of function mutants, lignin and cellulose contents were reduced [15]. Similarly, deficiency in cinnamoyl-CoA reductase (CCR) was identified in the *Arabidopsis irx4* mutant, which showed a collapsed xylem due to irregular and 50% lower lignin deposition in the stalk, compared to wild type [14]. However, stalks with a certain degree of flexibility exhibit increased tensile strength, which improves the resistance to stalk fracture [4, 16], and is thus favorable for crop improvement and production. However, the studies on such kind of flexibility in maize stalk have been limited to date.

Long-term field observations have indicated that the stalk of the maize inbred line JING724 tends to break at the first internode above the ear when exposed to strong wind before tasseling [17]. In contrast, the stalk of its derived line JING724A1 is more flexible, with improved wind resistance. Here, we developed a test to quantify the stalk fracture angle and used it to evaluate the flexibility of the stalk, and then systematically examined the mechanisms underlying flexibility in the stalk architecture, vascular bundle arrangement and development, and cell wall composition for both inbred lines JING724 and JING724A1 before tasseling. We used bulk segregant analysis (BSA) of their F_2:3_ population to identify one QTL with two candidate genes contributing to stalk flexibility in maize. Our findings provide valuable insights into the molecular and physiological mechanisms governing stalk fracture and lodging in maize, which will facilitate the improvement of lodging resistance in maize varieties.

## Results

### JING724 exhibits greater stiffness while JING724A1 is more flexible

**Fig. 1:**
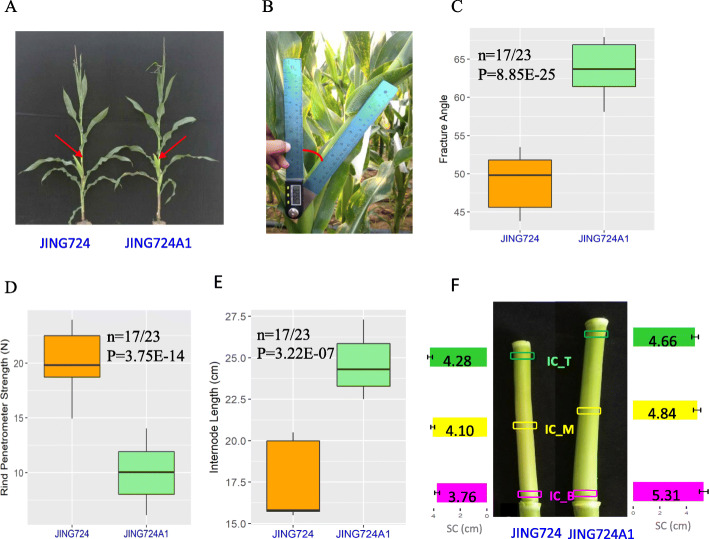
Stalk architecture and characteristics associated with stalk fracture in JING724 and JING724A1. **a** The plant architecture of JING724 and JING724A1. The position indicated by the arrow represents the first internode above the ear where the stalk is easily broken. **b** The measurement of stalk fracture angle. **c** Stalk fracture angle of the two lines. **d** The rind penetrometer strength of the two lines. **e** The length of the internode. **f** The stalk circumference (SC) at the bottom (IC_B), middle (IC_M), and top (IC_T) of the stalk with the sample sizes as 20 for both lines

The inbred line of JING724A1 was selected from the BC_1_F_4_ population derived from a cross between JING724 and the pollen donor MC01 (Fig. S1), so it shared similar genetic background to JING724 (Fig. 1a). In order to evaluate the mechanical strength of each line, we measured the stalk fracture angle and the rind penetrometer strength at the first internode above the ear before tasseling for JING724 and its derived line JING724A1. The stalk breakage for both lines occurred in the basal zone (BZ) of this internode. The fracture angle of JING724 was 48.96°, significantly smaller than 63.55° of JING724A1 (Fig. 1b-c), thus indicating higher flexibility of JING724A1. However, the rind penetrometer strength of JING724A1 was 9.99 N, which was significantly lower than 19.96 N recorded for JING724 (Fig. 1d), indicating a stiffer stalk for the JING724 line. Our results implied a potentially negative correlation between stalk stiffness and stalk flexibility.

### JING724A1 has longer and thicker internodes

In order to explore the relationship between the stalk architecture and its mechanical properties, and to identify differences in these properties between cultivars, the lengths and circumferences were measured at the internode above the ear. The internode lengths were 18.66 cm and 22.02 cm for JING724 and JING724A1, respectively (Fig. 1e). The average circumference of the base (IC_B), middle (IC_M), and top (IC_T) of the internode in JING724 was 3.76 cm, 4.1 cm, and 4.28 cm, respectively (Fig. 1f), whereas the average measurements were 5.31 cm, 4.84 cm and 4.67 cm at the same respective positions in JING724A1. The circumferences at all three positions for JING724A1 were significantly greater than those of JING724. Furthermore, the internode of JING724A1 was gradually tapered from the base to the top, while JING724 exhibited the opposite morphology, with a greater circumference at the top of the internode, suggesting that differences in stalk architecture may substantially impact the wind resistance of the two maize lines. In light of our findings of the higher flexibility of JING724A1, we speculate that the conical frustum shape of the stalk architecture enables higher wind resistance.

### JING724A1 has more vascular bundles and thick sclerenchyma cells

**Fig. 2:**
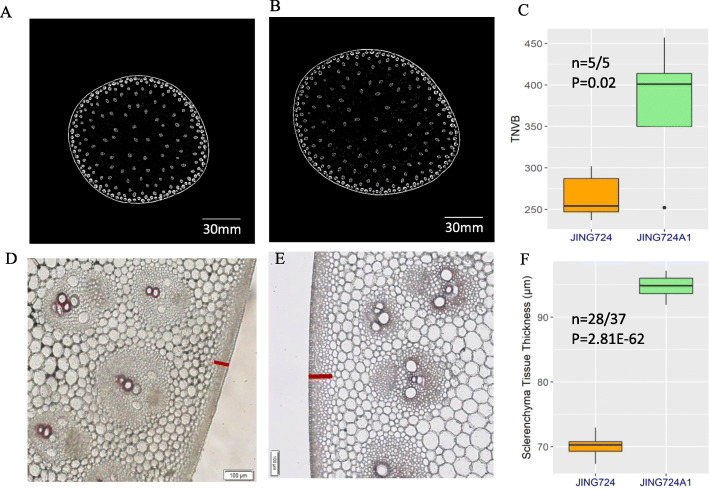
The vascular anatomy of JING724 and JING724A1. X-ray microcomputed tomography scans of **a** JING724 and **b** JING724A1. The scale bar of 30 mm is at the bottom right of each figure. **c** The total number of vascular bundles (TNVB) of the two lines. Phloroglucinol-stained transverse sections of stalk from **d** JING724 and **e** JING724A1. The red bars indicate the thickness of sclerenchyma tissue. The scale bar of 100 μm has been marked in each figure. **f** Quantitative comparison of sclerenchyma thickness between the two lines

To better understand the internal anatomical differences that potentially contribute to differences in stalk structure between JING724 and JING724A1, we employed X-ray microcomputed tomography to examine the development and total number of vascular bundles in both lines. Cross sections of JING724A1 stalk revealed an average of 474.8 vascular bundles, which was significantly more than the average of 265.4 observed in JING724 (Fig. 2a-c).

In addition, anatomical observation by optical microscopy showed that the structure of vascular bundles was highly similar between varieties, the staining was shallow, indicating that the vascular bundles were still immature with limited lignin accumulation at this stage (Fig. 2d-e). The thickness of sclerenchyma cells averaged 94.64 μm in JING724A1, which was significantly thicker than corresponding cells of JING724 which averaged 70.04 μm (Fig. 2f). In addition, we can see that the sclerenchyma tissue of JING724A1 was much looser than that of JING724. Taken together, these data suggest that the number of vascular bundles and the sclerenchyma thickness share a positive relationship with stalk fracture angle, and thus stalk flexibility.

### JING724A1 has significantly more glucose and different proportions of other cell wall components compared to JING724

**Fig. 3:**
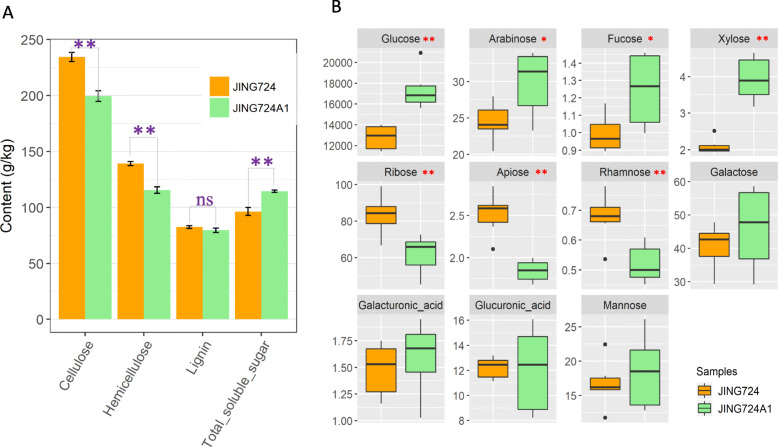
Differences in cell wall composition. **a** Variation in cellulose, hemicellulose, lignin, and total soluble sugars between JING724 and JING724A1. **b** Differences in the content of eleven monosaccharides in stalks of JING724 and JING724A1. * and ** denote significance at *p*-values less than 0.05 and 0.01, respectively, and ns means no significant difference

To clarify the function of different cell wall components in stalk flexibility, we measured cellulose, hemicellulose, lignin, and total soluble sugar contents in both maize inbred lines. Both cellulose and hemicellulose contents in JING724 were significantly higher than those of JING724A1, although the total soluble sugar content was higher in JING724A1 (Fig. 3a). Interestingly, no significant difference in lignin content was observed between the two lines. The proportion of total soluble sugar in JING724A1 was 18.81% higher than that of JING724 (Fig. S3), while cellulose, hemicellulose, and lignin components were 16.98, 14.94, and 3.57% lower in JING724A1, respectively.

We subsequently detected the levels of 11 types of monosaccharides in the stalks of JING724 and JING724A1 (Fig. 3b). Glucose, in particularly, can be accounted for 98% of the total monosaccharide content, whereas fructose, xylose, apiose, rhamnose, and galacturonic acid were all very minor cell wall components. In JING724A1, ribose, raffinose, and apiose were significantly lower than in JING724, while glucose, arabinose, rhamnose, and xylose were all significantly higher than in JING724. Notably, glucose content was 4577.47 mg/kg higher in JING724A1 than in JING724, which contributed to the substantially greater total monosaccharide content.

These data cumulatively suggest that the flexibility of JING724A1 may be attributed to the longer, thicker, conical frustum shape internode, thickened but loose sclerenchyma tissue, an increased number of vascular bundles, and higher total soluble sugar, especially glucose content in the cell wall.

### QTL correlated with stalk fracture angle

**Fig. 4:**
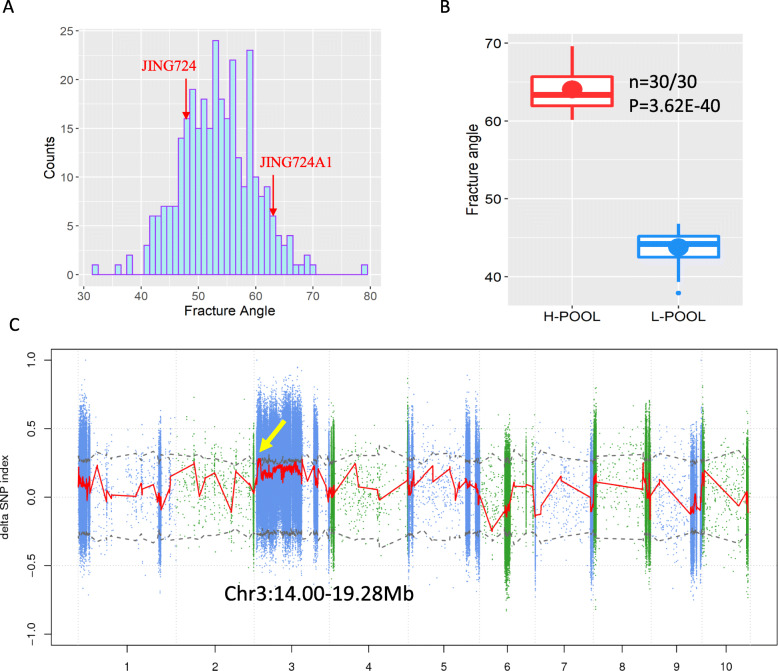
Stalk fracture angle and bulk segregant analysis (BSA) for the F_2:3_ population. **a** Histogram of the distribution of stalk fracture angles across the F_2:3_ population. The red arrows indicate the parental values. **b** Boxplot of the stalk fracture angles showing the difference between L-pool and H-pool lines used for BSA. **c** BSA results of stalk fracture angle. The X-axis represents the chromosome and the Y-axis represents the Δ (SNP-index). The blue and green dots indicate the SNPs; the red line is the regression line of the Δ (SNP-index) in each 1000 Kb window; the dark gray dashed line is the 95% threshold value; the region indicated by the yellow arrow is the putative QTL

In order to develop markers that can be used to select for decreased lodging in corn, it is necessary to identify genetic loci that underly quantitative differences in stalk flexibility. To this end, we measured stalk fracture angle before tasseling in 313 F_2:3_ families which each contained five plants derived from a JING724 and JING724A1 cross. The stalk fracture angles measured across the whole population ranged from 31.8° to 79.4°, with an average of 53.4° (Fig. 4a). Compared with the parental lines, 96 lines (30.86%) exhibited a fracture angle lower than JING724, while 17 (5.47%) fractured at greater angles than JING724A1, with the remaining 198 lines exhibited fracture angles intermediate to the parents.

The F_2_ plants corresponding to the 30 lowest (37.9°-46.8°) and 30 highest (60.13°-69.6°) stalk fracture angles were thus selected, and DNA from each set of 30 was combined into an L-pool and an H-pool, respectively (Fig. 4b). The DNA of the two parents and pools were used for BSA sequencing. A total of 243.55Gb high quality clean data were obtained by Illumina sequence for the two parents and pools. An average sequence depth was 21.74 x and 31.19 x for the two parents and the pools, respectively (Table S1). The high quality reads were mapped to the maize reference genome B73 RefGen_v4, with the average mapped rate of 98.54% for the two parents and 98.52% for the pools. An average of 5.22 M high quality SNPs and 0.71 M InDels (insertions and deletions) were revealed for the two parents; and 5.74 M SNPs and 0.64 M InDels were revealed for the pools (Table S2). There were an average of 92,911 SNPs and 6045 InDels with important functional mutations (nonsynonymous mutation, frameshift deletion, frameshift insertion, stopgain, stoploss). These variations were mainly concentrated in chr3:10–170 Mb, 180–200 Mb, 230–232 Mb and scattered on other chromosomes.

Using SNP-index association algorithms, two regions on chr3 of 14.00–14.70 Mb and 18.40–19.28 Mb were found above the threshold value at 95% confidence interval. Considering that the population used in this study was the F_2_ population, and the two intervals were relatively close to each other, we then performed linkage testing in the region between the two regions. Four KASP markers were developed in 16-18 Mb and showed significant correlation with the stalk fracture angles for the whole population (*p* < 0.05, Table S3). Therefore, we merged these three adjacent regions into a complete QTL interval as chr3:14.00–19.28 Mb. There were 1349 SNPs and 942 InDels in this interval, including 124 genes (Fig. 4b).

### Identification of genes potentially associated with the fracture angle trait

In order to further identify the candidate genes in this interval, we used RNA-Seq technology to obtain the gene expression at the basal zone of the first internode above the ear of the parents before the tasseling stage. For the 124 genes in the QTL interval, we found that 52 genes were expressed in the stalks of both parents, and only 12 genes were differentially expressed (− 1 < log_2_FC < 1; Table S4). Furthermore, Δ (SNP-index) value were used to spot the candidate genes. We found that among the 12 genes, only 2 genes whose Δ (SNP-index) exceeded the corresponding window threshold, namely Zm00001d039769 and Zm00001d039913.

**Fig. 5:**
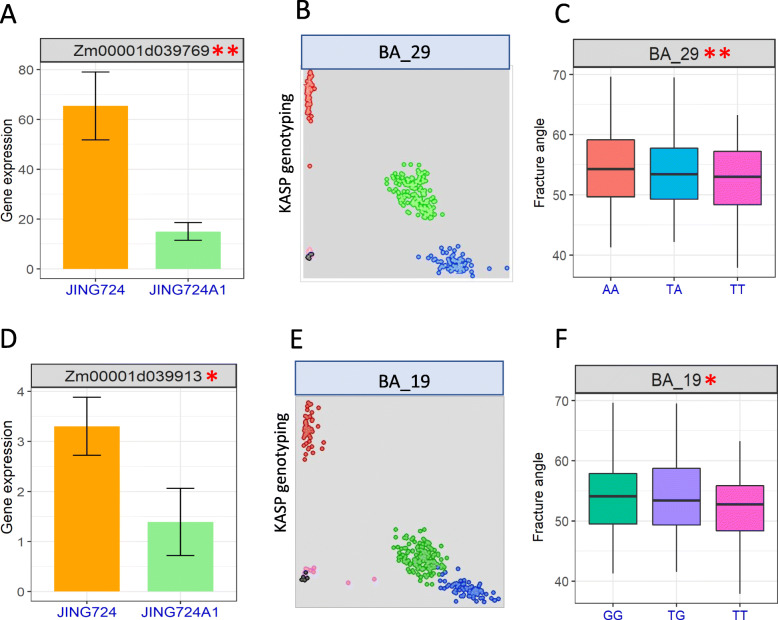
The relative expression levels of candidate genes and their KASP markers. **a-c** The expression and KASP result of Zm00001d039769. **d-f** The expression and KASP result of Zm00001d039913. **a** and **d** The relative expression levels of candidate genes for the two parents. Three independent experiments each using three biological replicates were used to generate these data. **b** and **e** Genotyping with KASP markers designed for each candidate gene. **c** and **f** The genotype of two KASP markers and their respective associations with fracture angle phenotype in the F_2:3_ population. * and ** denote significance at the level of 0.05 and 0.01, respectively

Zm00001d039769 was located on chr3: 14333996–14,339,635, containing 6 exons, and encoded a RING/U-box superfamily protein. Its expression level in JING724 was significantly higher than that in JING724A1, with the log_2_FC of 1.28 (Fig. 5a). BSA data revealed that there were 117 SNVs (SNP and indel) in this gene. Of these, 13 important mutations (non-synonymous mutations, non-frameshift insertion) located on the 3rd, 4th, and 6th exon resulted in the changes of protein sequence (Table S5). And there were 91 SNVs located in the upstream, 5’UTR, introns, 3’UTR, and downstream which may cause the changes of gene expression. Besides, there were 13 SNVs with synonymous mutations in the exons.

Zm00001d039913 was located on chr3: 19194748–19,210,804, containing 5 short exons and long introns, and annotated as a MADS-box transcription factor 54 (*mads54*). The expression level of this gene in JING724 was significantly higher than that of JING724A1, with the log_2_FC of 1.65 (Fig. 5a). BSA analysis showed that there were 304 SNVs in this gene, including 7 protein sequence changing mutations (stopgain, nonsynonymous, non-frameshift insertion) on the 2nd, 3rd and 5th exon (Table S5). And there were 294 SNVs located in the upstream, 5’UTR, introns, 3’UTR, and downstream which may cause the changes of gene expression. In addition, there were 3 SNVs with synonymous mutations in the exons.

To verify the association of these two genes with stalk fracture angle, we designed Kompetitive Allele-Specific PCR (KASP) markers for the SNPs at chr3:14338239 and chr3: 19204088 that caused non-synonymous mutations in Zm00001d039769 and Zm00001d039913, respectively, and performed genotyping for the whole F_2_ population (Table S6). Both of the markers were significantly correlated with the stalk fracture angle (*p* < 0.05) (Fig. 5b-c), further indicating that the two genes were potentially associated with the fracture angle trait. The two KASP markers might be applied in future studies for marker-assisted selection for stalk flexibility. Functional characterization of the two candidate genes will be carried out in the coming research.

## Discussion

Lodging due to stalk fracture can seriously interfere with the mechanical harvesting process of corn, and hence negatively impact yield [1]. Although many previous studies have used stalk stiffness as an indicator for resistance to lodging in crop plants, flexibility may be an indispensable trait in some cases [18, 19]. Generally, the stalk breaks when wind pressure exceeds the load that a plant can withstand. To adapt to a range of wind conditions, some severe and unpredictable, plants have evolved numerous traits favorable to survival in high wind, especially changes in cell wall chemical composition and stalk architecture, or structural morphology, at all scales, ranging from the cellular level to the whole plant [20]. In this study, using two genetically similar inbred maize lines, we found several traits that affect stalk flexibility. We also used bulk segregant analysis and QTL mapping to identify two candidate genes potentially associated with stalk flexibility.

### Stalk architecture affects flexibility

In this study, the stalk of the more flexible line, JING724A1, was thicker at the basal position of the first internode above the ear than JING724 (Fig. 1c), the more brittle line. This finding was consistent with previous studies demonstrating that a thicker stalk enhances lodging resistance [11, 21]. In addition, we observed a conical frustum shape of internode of JING724A1 but an inverted frustum of JING724 (Fig. 1d). Previous work has reported that the base of the internode is more susceptible to rupture than the middle zone [17]. Therefore, a less-developed and slender base may lead to poor lodging resistance. We hypothesize that the thick base and conical frustum internode provide an advantage of robust wind resistance. Moreover, we found that the number of vascular bundles and the thickness of the sclerenchyma in JING724A1 were significantly greater and looser than those in JING724, which likely contributes to the greater stalk flexibility in JING724A1 (Fig. 2). These results were in the agreement with previous works [3–5].

### Total soluble sugars and glucose content in the stalk cell walls affect flexibility

Our data revealed a substantial decrease in cellulose and hemicellulose of JING724A1 stalks compared to those of JING724, which were inconsistent with the cell wall composition in all other previously characterized fragile stalk mutants [3–8, 12, 13]., However, the total soluble sugars in JING724A1 stalks were significantly higher than the brittle line JING724 (Fig. 3a). Among the total soluble sugars, we found that glucose content contributed the largest difference in cell wall composition between lines, and JING724 had less glucose than JING724A1, which was in agreement with the maize brittle stalk *bk4* in which the glucose content was significantly lower than that in wild type [8]. Glucose can be present as a free monosaccharide or as a structural carbohydrate, polymerizing to form cellulose and hemicellulose in the stalk [22–24]. Taken together, these data suggest that disruption in the balance between structural and non-structural glucose can result in changes in cell wall composition and stalk flexibility, with properties of stalk flexibility attributable to an elevated content of non-structural glucose.

### Bulk segregant analysis reveals genes potentially associated with stalk fracture angle

Given that BSA is a fast, effective, and established method for identifying QTLs [25], we used this technique to map a novel quantitative trait of stalk fracture angle. Then the gene expression together with the ∆ (SNP-index) were used to narrow down the QTL region. Fortunately, we have identified two candidate genes that contain important mutations, and their expression levels were significantly different between the parents. One gene Zm00001d039913 is annotated as a MADS-box transcription factor 54, which has been reported to function in floral development [26]. The other gene Zm00001d039769 encodes a RING/U-box superfamily protein, which has been shown to regulate asymmetric division and proliferation of cells in the apical meristem of *Arabidopsis* [27]. Previous studies have shown that RING/U-box proteins may serve as the E3 ligase domain in the ubiquitination pathway mediating DELLA protein degradation, as part of the response to gibberellic acid [28]. Gibberellic acid (GA) has been widely reported to control internode length in rice and maize [29, 30]. This background led us to speculate that the differential expression of Zm00001d039769 points to its participation in the GA pathway-mediated regulation of internode architecture and hence determination of the stalk fracture angle.

## Conclusions

Based on our study, we conclude that (1) The stalk fracture angle can be used to reflect the stalk flexibility and the lodging resistance of the stalk. (2) The stalk with better flexibility had several characteristics: a longer, thicker stalk and with a conical, frustum-shaped internode; more vascular bundles; thicker but looser sclerenchyma tissue; more total soluble sugar, especially more glucose component. (3) One QTL region located on chr3:14.00–19.28 Mb was detected for stalk fracture angle. (4) The candidate genes were Zm00001d039769 and Zm00001d039913, which exhibited significantly different levels of transcriptional expression between parents, and contained important variations. (5) Two KASP markers were designed for the two genes. The traits related to the flexible stalk and the identification of potential genes provide new insights into the mechanisms governing wind resistance and stalk fracture. This study provides a foundation to hasten the development of functional molecular markers for marker-assisted breeding of maize varieties with enhanced resistance to lodging.

## Methods

### Plant materials

The two inbred maize lines, JING724 and JING724A1, used in this study were developed by the Maize Research Center, Beijing Academy of Agriculture and Forestry Sciences (Beijing, PRC, Fig. 1a). JING724 was the female parent of the hybrid Jingke968, one of the leading maize varieties in China. JING724A1 was the female parent of the hybrid Jingke968A, which was bred for stronger resistance to stalk fracture than Jingke968. JING724A1 was selected from the BC_1_F_4_ population derived from a cross between JING724 and the pollen donor MC01, which shared more than 80% genetic similarity with JING724 (Fig. S1, Additional file 2). In order to locate the QTL, the two materials were crossed and then self-pollinated to F_2_ population. Each F_2_ individual was self-pollinated to obtain each F_2:3_ family, and finally a total of 313 F_2:3_ families were produced (Fig. S1). The phenotypic data related to stalk fracture for the JING724A1 and JING724 inbred lines and their F_2:3_ families were collected in the Hainan research station during the winter of 2017 and 2018. DNA from each F_2_ individual was extracted using the CTAB method.

### Mechanical strength measurements

The mechanical strength of the stalk was evaluated by two methods, the rind penetrometer strength and the stalk fracture angle, which were then used to evaluate the stalk stiffness and flexibility, respectively. The rind penetrometer strength was determined at the basal zone (BZ) using a YYD-1 instrument (Zhejiang Top Cloud-Agri Technology Co., Ltd., Zhejiang, China), which mainly uses a pressure sensor with the diameter of 1 mm to detect the puncture strength of the rind [17].

For measurement of the stalk fracture angle, we used a modified YYD-1 instrument with a JL-360-01 digital angle ruler (Xin Liang Instrument Technology Co., Ltd., Shanghai, China) (Fig. S2). To ensure that measurement position was consistent, a 20 cm rope was tied to the probe of the YYD-1 sensor, with the other end of the rope fixed to the stalk at 60 cm above the first ear, with the node at the first ear manually immobilized. A constant force was then applied to the sensor to horizontally pull the stalk until it broke, and the angle between the original and broken positions was recorded as the stalk fracture angle. Since the measurement of the stalk fracture angle was destructive to the plants, the phenotypic scores for the corresponding F_2_ were the mean angle value of five individual plants randomly selected from each F_2:3_ family.

### Internode architecture measurement

Twenty plants at the same growth stage were selected from each line, JING724 and JING724A1, to measure the architecture of the first internode above the ear, including the internode length and the internode circumference. The positions measured for the internode circumference were established by Zhang [31], including the internode circumference of the basal zone (IC_B); the internode circumference of the middle of the internode (IC_M); and the internode circumference of the top of the internode (IC_T). The stalk circumference can be obtained by using a soft ruler.

### X-ray microcomputed tomography and optical microscopy observation

Five replicate stalk cross sections, 5 mm thick, sampled at the BZ were collected from both JING724 and JING724A1. The total number of vascular bundles (TNVB) in each stalk cross section was determined using X-ray microcomputed tomography (CT) [17, 32].

For microscopic observations, stalk thin sections (< 1 mm thick) were sampled from the BZ of each cultivar with a scalpel, then immediately stained with phloroglucinol solution (2%). Ten samples for each maize line were observed, with three fields for each sample observed at 100X magnification using an Olympus DP80 compound microscope (Olympus Corp., Shinjuku, JP). The thickness of sclerenchyma tissue was measured by the Image-Pro Plus 6.0 software [33].

### Analysis of stalk cell wall components

To determine the cell wall composition of each maize line, 3 BZ samples were collected from each of ten biological replicates for both JING724 and JING724A1. The concentrations of cellulose, hemicellulose, and lignin were measured and calculated using a high performance liquid chromatography system (1260 series, Agilent Technologies, Santa Clara, CA, USA) as previously described [17]. The total soluble sugars were extracted with distilled water and the concentration for each sample was determined by the anthrone/H_2_SO_4_ method using a UV-VIS spectrometer (TU-1901, Beijing Purkinje Instruments Co. Ltd., Beijing, China) [34]. For detection of monosaccharides, six samples from each of three biological replicates were collected from each inbred line. The monosaccharides in samples were labeled with 1-phenyl-3-methyl-5-pyrazolone, according to protocols described by Honda [35]. The reaction products were dried, then re-dissolved thoroughly in methanol. The supernatants were used to detect monosaccharides by UHPLC-HRMS (ultra-high-performance liquid chromatography coupled to high-resolution mass spectrometry) [36]. Briefly, 2 μl of each sample were injected into a 100 × 2.1 mm × 1.7 μm BEH C18 LC column (Waters Corp., Milford, MA) [37] with phase A comprised of 0.1% formic acid aqueous solution, phase B of 0.1% formic acid acetonitrile solution, and a column temperature of 45 °C. Mass spectrometry was performed in full scan mode using a XEVO G2XS Q-TOF high resolution mass spectrometer (Waters Corp., Milford, MA). The ion source parameters were as follows: capillary voltage = 2000 V, sampling cone = 40 V, source temperature = 115 °C, desolvation temperature = 500 °C, desolvation gas = 900 L/h, scan range = 400 ~ 600, scan time = 0.3 s.

Solutions containing standards for each monosaccharide were also analyzed to establish a standard curve. For each target compound, the derivatized parent ion was used for detection, and concentration (mg/kg) was determined using the standard curve.

### Bulk Segregant Analysis (BSA) for stalk fracture angle

In the F_2:3_ population, the top 30 lines with the greatest and smallest stalk fracture angles were selected to construct high bulk (H) and low bulk (L) sequencing pools, respectively. DNA from each plant was individually extracted and pooled together in equal concentrations, with the total amount of DNA in each pool > 3 μg. Paired-end libraries with an insert size of 500 bp were constructed using a Paired-End DNA Sample Prep kit (Illumina Inc., San Diego, CA, USA), and were sequenced using the HiSeq X10 (Illumina Inc., San Diego, CA, USA) NGS platform by Genedenovo (Guangzhou, China). Clean reads were mapped to the maize reference genome B73_RefGen_v4 (https://www.ncbi.nlm.nih.gov/assembly/GCF_000005005.2/) using BWA software with the settings of ‘mem 4 -k 32 -M’ [38]. Variant calling was performed using the GATK Unified Genotyper. SNPs and InDels were filtered using GATK Variant Filtration with proper settings (−Window 4, −filter “QD < 4.0|| FS > 60.0 || MQ < 40.0 “, −G_filter “GQ < 20”). The variants exhibiting segregation distortion or sequencing errors were discarded. The final variants were annotated using ANNOVAR software [39].

BSA was carried out following the method described by Takagi [40] with JING724A1 as a reference genome and JING724 as the mutant parent. A 1000 kb sliding window with a 100 kb step size were employed for calculating the SNP-index of the offspring pools, and calculate the ∆ (SNP-index) with the difference between the SNP-index of the two offspring pools. The threshold at the confidence level of 95% was determined by 1000 permutations for each window. Peaks that exceeded the threshold were considered as candidate QTL regions, and the candidate genes harbored in these regions were identified according to their annotation, SNP/InDel mutation, and transcription level.

### The development of Kompetitive Allele-Specific PCR (KASP) markers

To develop the KASP markers for the important SNP, allele-specific forward primers and a common reverse primer were designed based on the sequence of JING724 using BatchPrimer3 (https://probes.pw.usda.gov/batchprimer3/) [41]. The KASP primers mixture containing Primer_AlleleFAM, Primer_AlleleHEX, and Primer_Common were synthesized by LGC (LGC Genomics Ltd. Hoddesdon, UK). The total KASP reaction volume was 1ul (using 1536 microplates), including 30 ng of lyophilized DNA, 0.5 μl of KASP 2x Master Mix (KBS-1016-011, LGC Genomics Ltd. Hoddesdon, UK), 0.486 μl of ultrapure water, and 0.014 μl KASP primer mixture. The amplification program was as follows: 95 °C for 15 min; ten cycles of 94 °C for 20s and touchdown starting at 61 °C for 60s until to 55 °C, then 32 cycles of 94 °C for 20s and 55 °C for 60s. The fluorescence was scanned by Pherastar (LGC Genomics Ltd. Hoddesdon, UK), and the genotyping results were visualized and output files generated using Kraken software (LGC Genomics Ltd. Hoddesdon, UK).

### Transcriptome sequencing and data analysis

Transcriptome sequencing was performed on whole RNA extractions of BZ samples collected before tasseling. RNA extractions were performed on three independent experiments each using three biological replicates for both JING724 and JING724A1. The RNA extraction, library construction, sequencing, and data analysis were performed as previously described by Wang [17]. Briefly, the whole process was conducted as follows: RNA was extracted following the standard protocol for a Quick RNA isolation Kit (Huayueyang Biotechnology Co., Ltd. Beijing, China). Sequencing libraries were generated using NEBNext Ultra™ RNA Library Prep Kit for Illumina (NEB, USA). The libraries were sequenced using an Illumina HiSeq™ 2000. Low-quality sequence reads were removed from the datasets. The paired-end reads were mapped onto the B73 AGPv3.25 reference using Hisat2 tools software. Unigene annotation was performed using BLAST software [42]. Gene expression levels were estimated by fragments per kilobase of transcript per million fragments mapped reads (FPKM) [43].

### Statistical analysis

Statistical analysis and data plotting were conducted with R3.4.4 software (https://cran.r-project.org/bin/windows/base/old/3.4.4/), and student’s t-test was used to determine significant differences.

## Supplementary Information

**Additional file 1:** **Figure S1.** The construction process of JING724A1 and the F_2:3_ population. (A) The development of JING724A1. (B) The construction of the F_2:3_ population using JING724 and JING724A1. **Figure S2.** Measurement of stalk fracture angle. **Figure S3.** The increased ratio of specific cell wall components in JING724A1 compared to JING724. **Table S1.** Overview of sequencing results. **Table S2.** Summary of SNPs and InDels for the parents and pools. **Table S3.** Four KASP makers and the significance with the stalk fracture angle. **Table S4.** The expression level of 12 genes. **Table S5.** The important SNPs and InDels for the two candidate genes. **Table S6.** Sequences of two KASP markers for the two candidate genes.

**Additional file 2.** The genetic information of JING724 and MC01 revealed by 1212 SNPs and 60 K chip.

## Data Availability

All the data on the present study has been included in the tables or supplementary tables in this manuscript. The datasets used and/or analyzed in this study are available from the corresponding author on reasonable request.

## Abbreviations

BZ: Basal zone
IC_B: Internode circumference of the basal zone
IC_M: Internode circumference of the middle of the internode
IC_T: Internode circumference of the top of the internode
TNVB: Total number of vascular bundles
CT: X-ray microcomputed tomography
UHPLC-HRMS: Ultra-high-performance liquid chromatography coupled to high-resolution mass spectrometry
BSA: Bulk Segregant Analysis
KASP: Kompetitive Allele-Specific PCR
InDel: Insertion and deletion
