# Supplementary material for: Stalk architecture, cell wall composition, and QTL underlying high stalk flexibility for improved lodging resistance in maize
Source: BMC Plant Biol. 2020 Nov 11;20:515. doi: 10.1186/s12870-020-02728-2 (PMC7659129; doi:10.1186/s12870-020-02728-2)
Supplement: Supplementary file 1 — Additional file 1: Figure S1. The construction process of JING724A1 and the F2:3 population. (A) The development of JING724A1. (B) The construction of the F2:3 population using JING724 and JING724A1. Figure S2. Measurement of stalk fracture angle. Figure S3. The increased ratio of specific cell wall components in JING724A1 compared to JING724. Table S1. Overview of sequencing results. Table S2. Summary of SNPs and InDels for the parents and pools. Table S3. Four KASP makers and the significance with the stalk fracture angle. Table S4. The expression level of 12 genes. Table S5. The important SNPs and InDels for the two candidate genes. Table S6. Sequences of two KASP markers for the two candidate genes. [file 12870_2020_2728_MOESM1_ESM.docx]

**SUPPLEMENTARY INFORMATION**

**Stalk architecture, cell wall composition, and QTL underlying high stalk flexibility for improved lodging resistance in maize**

Xiaqing Wang^1,3^, Zi Shi^1,3^, Ruyang Zhang^1^, Xuan Sun^1^, Jidong Wang^1^, Shuai Wang^1^, Ying Zhang^2^, Yanxin Zhao^1^, Aiguo Su^1^, Chunhui Li^1^, Ronghuan Wang^1^, Yunxia Zhang^1^, Shuaishuai Wang^1^, Yuandong Wang^1^, Wei Song^1*^, Jiuran Zhao^1*^

*^1^Beijing Key Laboratory of Maize DNA Fingerprinting and Molecular Breeding, Maize Research Center, Beijing Academy of Agriculture & Forestry Sciences (BAAFS), Shuguang Garden Middle Road No. 9, Haidian District, Beijing 100097, China.*

*^2^Beijing Key Lab of Digital Plant, Beijing Research Center for Information Technology in Agriculture, Beijing Academy of Agriculture and Forestry Sciences (BAAFS), Shuguang Garden Middle Road No. 11, Haidian District,* *Beijing 100097, China.*

*^3^These authors contributed equally to this work.*

*^*^*Corresponding authors:

Wei Song, Tel: (+86) 01051503983， E-mail: [songwei1007@126.com](mailto:songwei1007@126.com)

Jiuran Zhao, Tel: (+86) 01051503936，E-mail: [maizezhao@126.com](mailto:maizezhao@126.com)


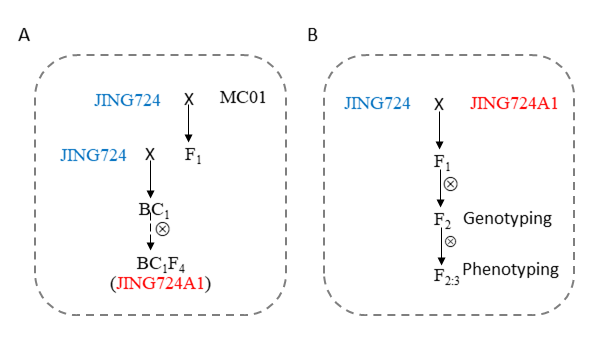


**Fig. S1** The construction process of JING724A1 and the F_2:3_ population. (A) The development of JING724A1. (B) The construction of the F_2:3_ population using JING724 and JING724A1.


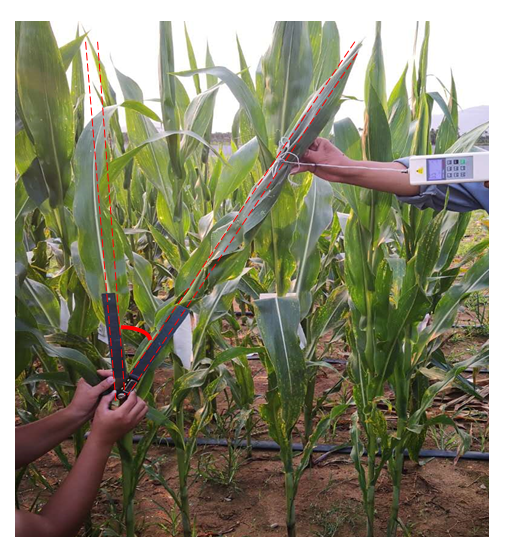


**Fig. S2** Measurement of stalk fracture angle.


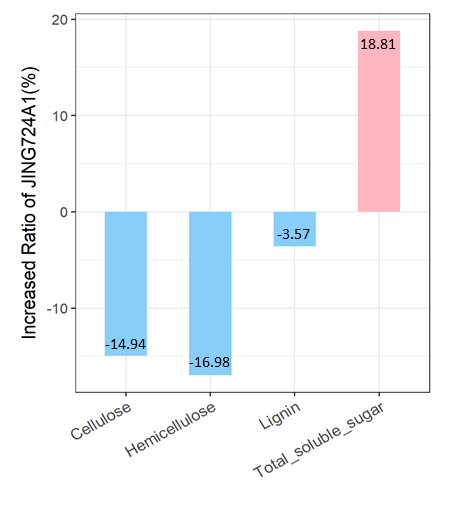


**Fig. S3** The increased ratio of specific cell wall components in JING724A1 compared to JING724.

**Table S1** Overview of sequencing results.

| Sample | Clean reads | High quality  clean data (bp) | Q30 (%) | GC (%) | Properly mapped (%) | Average depth |
| --- | --- | --- | --- | --- | --- | --- |
| H-pool | 505,846,216 | 75,588,837,622 | 91.16% | 47.82% | 98.71% | 29.95X |
| L-pool | 454,234,782 | 67,921,209,847 | 90.73% | 47.08% | 98.36% | 26.79X |
| J724 | 357,343,896 | 53,463,500,489 | 90.81% | 46.98% | 98.55% | 21.11X |
| J724A1 | 311,254,540 | 46,576,669,927 | 90.37% | 47.64% | 98.46% | 18.30X |

**Table S2** Summary of SNPs and InDels for the parents and pools.

| Sample | SNPs | InDels |
| --- | --- | --- |
| H-pool | 5,760,595 | 710,352 |
| L-pool | 5,725,864 | 707,044 |
| JING724 | 5,277,813 | 652,925 |
| JING724A1 | 5,158,743 | 636,008 |

**Table S3** Four KASP makers and the significance with the stalk fracture angle.

| Marker | Chr | Location | Ref | Mut | p-value |
| --- | --- | --- | --- | --- | --- |
| KA_1 | 3 | 16,414,171 | C | T | 0.02 |
| KA_2 | 3 | 16,468,608 | C | G | 0.02 |
| KA_3 | 3 | 16,736,281 | T | G | 0.03 |
| KA_4 | 3 | 17,544,848 | T | G | 0.04 |

**Table S4** The expression level of 12 genes.

| Gene_ID | Chr | Start | End | log_2_FC |
| --- | --- | --- | --- | --- |
| Zm00001d039769 | 3 | 14,333,996 | 14,339,635 | -1.28 |
| Zm00001d039824 | 3 | 15,918,978 | 15,922,548 | 1.71 |
| Zm00001d039825 | 3 | 15,958,238 | 15,958,636 | -2.82 |
| Zm00001d039827 | 3 | 15,992,310 | 15,992,768 | 3.03 |
| Zm00001d039837 | 3 | 16,287,892 | 16,290,203 | -1.07 |
| Zm00001d039848 | 3 | 16,535,244 | 16,539,074 | 1.31 |
| Zm00001d039859 | 3 | 16,955,374 | 16,956,401 | 2.14 |
| Zm00001d039880 | 3 | 17,453,267 | 17,454,450 | -4.9 |
| Zm00001d039882 | 3 | 17,546,111 | 17,550,377 | 1.9 |
| Zm00001d039891 | 3 | 18,182,217 | 18,183,569 | -4.73 |
| Zm00001d039893 | 3 | 18,371,543 | 18,374,234 | 1.17 |
| Zm00001d039913 | 3 | 19,194,748 | 19,210,804 | -1.65 |

**Table S5** The important SNPs and InDels for the two candidate genes.

| Gene | Chr | Start | End | Ref | Mut | Type | ΔSNP-index | Function type | Function effect* |
| --- | --- | --- | --- | --- | --- | --- | --- | --- | --- |
| Zm00001d039769 | 3 | 14,337,550 | 14,337,550 | G | A | SNP | 0.31 | nonsynonymous | T005:exon3:c.G280A:p.G94S |
| Zm00001d039769 | 3 | 14,337,614 | 14,337,614 | T | C | SNP | 0.22 | nonsynonymous | T005:exon3:c.T344C:p.V115A |
| Zm00001d039769 | 3 | 14,337,635 | 14,337,635 | G | T | SNP | 0.35 | nonsynonymous | T005:exon3:c.G365T:p.R122I |
| Zm00001d039769 | 3 | 14,337,643 | 14,337,643 | G | T | SNP | 0.30 | nonsynonymous | T005:exon3:c.G373T:p.A125S |
| Zm00001d039769 | 3 | 14,337,719 | 14,337,719 | G | A | SNP | 0.42 | nonsynonymous | T005:exon3:c.G449A:p.S150N |
| Zm00001d039769 | 3 | 14,337,754 | 14,337,754 | G | A | SNP | 0.53 | nonsynonymous | T005:exon3:c.G484A:p.A162T |
| Zm00001d039769 | 3 | 14,337,817 | 14,337,817 | A | G | SNP | 0.56 | nonsynonymous | T005:exon3:c.A547G:p.I183V |
| Zm00001d039769 | 3 | 14,338,006 | 14,338,006 | T | G | SNP | 0.13 | nonsynonymous | T005:exon3:c.T736G:p.L246V |
| Zm00001d039769 | 3 | 14,338,239 | 14,338,239 | A | T | SNP | 0.26 | nonsynonymous | T005:exon4:c.A796T:p.R266W |
| Zm00001d039769 | 3 | 14,338,307 | 14,338,307 | C | G | SNP | -0.03 | nonsynonymous | T005:exon4:c.C864G:p.H288Q |
| Zm00001d039769 | 3 | 14,338,476 | 14,338,476 | - | CCT | Insertion | - | nonframeshift insertion | T005:exon4:c.1033_1034insCCT:p.T345delinsTS |
| Zm00001d039769 | 3 | 14,338,719 | 14,338,719 | G | T | SNP | 0.16 | nonsynonymous | T005:exon4:c.G1276T:p.V426F |
| Zm00001d039769 | 3 | 14,339,220 | 14,339,220 | G | C | SNP | 0.24 | nonsynonymous | T005:exon6:c.G1551C:p.E517D |
| Zm00001d039913 | 3 | 19,204,088 | 19,204,088 | G | T | SNP | 0.48 | nonsynonymous | T001:exon2:c.G235T:p.V79F |
| Zm00001d039913 | 3 | 19,204,128 | 19,204,128 | G | T | SNP | 0.46 | nonsynonymous | T001:exon2:c.G275T:p.R92L |
| Zm00001d039913 | 3 | 19,204,134 | 19,204,134 | G | A | SNP | 0.44 | nonsynonymous | T001:exon2:c.G281A:p.C94Y |
| Zm00001d039913 | 3 | 19,204,178 | 19,204,178 | - | CATGCAGTTGTTATGAGTAAATATAAG | Insertion | - | stopgain | T001:exon2:c.325_326ins,p.S109delinsSCSCYEX |
| Zm00001d039913 | 3 | 19,209,490 | 19,209,490 | G | A | SNP | 0.31 | nonsynonymous | T002:exon3:c.G304A:p.D102N |
| Zm00001d039913 | 3 | 19,209,493 | 19,209,493 | G | A | SNP | 0.40 | nonsynonymous | T002:exon3:c.G307A:p.E103K |
| Zm00001d039913 | 3 | 19,210,504 | 19,210,504 | - | CGG | Insertion | - | nonframeshift insertion | T002:exon5:c.517_518insCGG:p.T173delinsTA |

* Note: Transcript: SNP location: coding sequence variation and its position: amino acid conversion and its position

**Table S6** Sequences of two KASP markers for the two candidate genes.

| ID | Gene | SNP | Primer_AlleleFAM | Primer_AlleleHEX | Primer_Common | Allele_FAM | Allele_HEX |
| --- | --- | --- | --- | --- | --- | --- | --- |
| BA_29 | Zm00001d039769 | Chr3:14338239 | GAAGGTGACCAAGTTCATGCTCCAACCACAGGATAGTAAGCCCT | GAAGGTCGGAGTCAACGGATTCCAACCACAGGATAGTAAGCCCA | CAGGTATGACTAGCAGGGTCCCAA | A | T |
| BA_19 | Zm00001d039913 | Chr3:19204088 | GAAGGTGACCAAGTTCATGCTATTCCTGTGTTTGAATGGATAAGTGAC | GAAGGTCGGAGTCAACGGATTAAATTCCTGTGTTTGAATGGATAAGTGAA | CATTCTATGACCTACATGAAACTAGGCAA | G | T |
